# The Interactive Effects of Nutrient Density and Breed on Growth Performance and Gut Microbiota in Broilers

**DOI:** 10.3390/ani14233528

**Published:** 2024-12-06

**Authors:** Meiting Jia, Jiaqi Lei, Yuanyang Dong, Yuming Guo, Bingkun Zhang

**Affiliations:** 1 State Key Laboratory of Animal Nutrition and Feeding, College of Animal Science and Technology, China Agricultural University, Beijing 100193, China; 2 College of Animal Science, Shanxi Agricultural University, Taiyuan 030800, China

**Keywords:** breed, nutrient density, growth performance, microbiome, broiler

## Abstract

This study investigated whether variations in growth response to low nutrient density across breeds are linked to microbiota regulation. Arbor Acres (**AA**) and Beijing-You (**BY**) were fed high- (**HN**) and low-nutrient (**LN**) diets from day (**d**) 0 to d42. Body weight, feed intake, and intestinal measurements were recorded, and microbiota from the ileum and cecum were analyzed on d7, d21, and d42. Results showed that AA broilers had greater growth performance with a lower feed conversion ratio (**FCR**) and greater average daily gain (**ADG**) than BY chickens. The LN diet negatively affected AA broiler growth due to impaired intestinal development, while BY chickens compensated by increasing feed intake. Microbiota composition was primarily affected by breed than by nutrient density, with AA broilers having more beneficial bacteria and BY chickens having more short-chain fatty acid (**SCFA**)-producing bacteria. The LN diets reduced anti-inflammatory bacteria such as *Shuttleworthia* and *Eisenbergiella* in the cecum on d7. By d21, LN diets decreased *Lactobacillus* and increased proinflammatory *Marvinbryantia*, potentially impairing growth. However, LN diets enriched SCFA-producing bacteria like *Ruminococcaceae_UCG.013*, *Eisenbergiella*, and *Tyzzerella* in BY chickens and *Faecalitalea* in AA broilers by d21, which may benefit gut health. By d42, LN diets reduced genera linked to intestinal permeability and fat deposition, including *Ruminococcus_torques_group*, *Romboutsia*, *Erysipelatoclostridium*, and *Oscillibacter*. Additionally, LN diets enriched *Christensenellaceae_R-7_group* in AA broilers, associated with intestinal barrier integrity, and increased anti-inflammatory bacteria *Alistipes* and *Barnesiella* in AA broilers and BY chickens, respectively, by d42. Overall, AA broilers were more susceptible to reduced nutrient density due to impaired intestinal development, while BY chickens adapted better by increasing feed intake. The microbiota responses to low nutrient density varied over time, potentially negatively affecting gut health in the early stage and growth in the middle stage but possibly improving lipid deposition and gut health in the middle and late stages.

## Simple Summary

In the poultry industry, dietary nutrient density plays a crucial role in promoting growth and maintaining health. However, feeding chicken excessive nutrient-rich diets can lead to poor intestinal health and lower meat quality. As feed costs rise, low-nutrient diets, which reduce energy and protein levels, have become a popular alternative, although they often result in slower growth, especially in fast-growing chicken breeds. This study aimed to compare how two different chicken breeds, Arbor Acres, a fast-growing breed, and Beijing-You, a slow-growing breed, respond to low-nutrient diets. It also examined the gut microbiota, which plays a vital role in digestion and gut health. The study found that Arbor Acres chickens were more affected by the low-nutrient diet, showing slower growth, while Beijing-You chickens adapted by increasing their feed intake. The differences in responses of gut bacteria to low-nutrient diets between the breeds may explain these varying growth responses. These findings can help inform effective feeding strategies that take into account a breed-specific response to nutrient density.

## 1. Background

In the broiler industry, high-nutrient (**HN**) diets are essential for maximizing growth potential and feed efficiency [1]. Diets rich in energy and crude protein meet the nutritional requirement for optimal growth and development. However, excessive high nutrient density may negatively affect intestinal health, increase fat deposition, and compromise meat quality, potentially limiting economic efficiency [2,3,4]. Given the high feed costs, low-nutrient (**LN**) diets, which lower crude protein and metabolizable energy levels, have gained significant attention.

Low-nutrient diets are often associated with increased feed intake and decreased body weight gain, particularly in faster-growing broiler breeds. For instance, Ahmadi-Sefat et al. [5] reported that a reduction of 100 kcal/kg in metabolizable energy (**ME**) or 5% in crude protein (**CP**) led to lower body weight and average daily gain in Ross-308 broilers. Similar results were observed in Cobb 400Y broiler, where a 3% reduction in ME diet increased feed intake and feed conversion rate (**FCR**) but decreased body weight gain [6]. However, slower-growing breeds demonstrated better adaptation to low-nutrient diets. Beijing-You (**BY**) chickens, a slow-growing native breed, require 91 days to reach a market weight of around 1.5 kg, whereas Arbor Acres (**AA**) broilers, a fast-growing commercial breed, typically reach a market weight of approximately 2.5 kg in 42 days. Zhao et al. [7] found that while reduced protein and energy diets significantly increased feed intake and decreased body weight gain in AA broilers, BY chickens exhibited less pronounced effects. Zhou et al. [8] also reported that dietary ME and crude protein levels did not affect growth performance in native-growing Danzhou chickens. These findings suggest that the faster-growing broilers are more vulnerable to reductions in nutrient density compared to the slower-growing breeds.

The commensal gut microbiota plays a crucial role in broiler growth performance by maintaining intestinal health, competing against pathogens, promoting villi development, and regulating the immune system [9,10]. Several studies have explored the relationship between dietary nutrient density and gut microbiota in chickens. For example, AA broilers fed a low-energy diet showed a decrease in the relative abundance of beneficial bacteria, such as *Tyzzerella* and *Candidatus_Arthromitus*, in the cecum by d21, and a decrease in lipid metabolism-related bacteria, such as *Erysipelatoclostridium* on d42 [11]. Cobb 500 broilers on a reduced crude protein diet exhibited higher levels of *Lactobacillus*, but lower levels of *Faecalibacterium* and *Erysipelatoclostridium* in the cecum [12]. In BY chickens, lower crude protein diets led to a reduction in *Proteobacteria*, a marker of microbiota dysbiosis [13]. Despite some consistent microbiota changes across breeds, the overall response of gut microbiota to nutrient response varied between breeds.

Although researchers have explored different responses of microbial communities to environmental challenges (such as heat stress) among distinct chicken breeds [14], studies on how microbial communities respond to low nutrient density and their relationship with growth performance sensitivity are relatively scarce. Furthermore, current research often focuses on specific gut segments or single time points, neglecting the impact of temporal dynamics on microbial community responses.

To better understand the role of genetic selection in gut microbiota response to low-nutrient diets, this study compared two chicken breeds with distinct growth rates. We aimed to investigate the interaction effects of breed and nutrient density on the intestinal microbiota at different time points and to explore the correlation between microbiota and growth performance. This study may help identify microbial communities associated with adaptation to reduced nutrient density, ultimately contributing to optimized nutritional strategies for broilers.

## 2. Methods

### 2.1. Animal Experiments and Sample Collection

All animal experimental procedures were approved by the Laboratory Animal Welfare and Ethics Committee at China Agricultural University. Day-of-hatch male Arbor Acres (**AA**) broilers (*n* = 192) and Beijing-You (**BY**) chicks (*n* = 192) were obtained from the Beijing Dafa Chia Tai hatchery (Shunyi district, Beijing, China) and the Beijing-You Breeding and Protection Base (Daxing district, Beijing, China), respectively. The initial average body weights were 43.06 g for AA broilers and 42.63 g for BY chickens. Chickens were reared at the Poultry Experimental Base of China Agricultural University (Zhuozhou, Hebei, China) for 42 days. The chicks were randomly assigned to two dietary treatments, including a high-nutrient (ME = 3053 kcal/kg; CP = 23%) diet and a low-nutrient (ME = 2900 kcal/kg; CP = 21%) diet (Table 1). Thus, there were four treatment groups: AA broilers fed the HN diet (AAHN) and the LN diet (AALN), and BY chicks fed the HN diet (BYHN) and the LN diet (BYLN). Each group had eight replicate cages with twelve chicks per cage (17 birds/m^2^). Chicks had ad libitum access to feed and water and were vaccinated according to guidelines. During the trial, feeding was carried out manually three times a day and water was provided using nipple drinkers. The lighting program consisted of 16 h of light and 8 h of darkness each day. Body weight, feed intake (**FI**), and mortality for each replicate cage were recorded on days (d) 7, d21, and d42 after fasting for 8 h to minimize variation caused by the weight of digestive tract contents. The average daily gain (**ADG**), average daily feed intake (**ADFI**), and feed conversion rate (**FCR**) were calculated. One chicken from each replicate cage was randomly selected, weighed, and euthanized for sample collection on d7, d21, and d42. Euthanasia was performed by electrocution followed by exsanguination, in accordance with ethical guidelines and animal welfare standards. The length of the duodenum, jejunum, ileum, and cecum were measured, and intestinal weights were measured after removing the intestinal contents. Approximately 1.5 g of digesta from the middle of the ileum and cecum was collected, snap-frozen in liquid nitrogen, and stored at −80 °C.

### 2.2. DNA Extraction and 16S rRNA Sequencing

Bacterial DNA in the digesta of the ileum and cecum was isolated using E. Z. N. A.^®^ Bacterial DNA Kits (Omega Bio-tek, Norcross, GA, USA). The quality and concentration of DNA samples were determined using agarose gel electrophoresis and a Nano-300 micro-spectrophotometer (Hangzhou Allsheng Instruments Co., Ltd., Hangzhou, China). Then, DNA samples were sent to Allwegene Technology Inc. (Beijing, China) for amplification and sequencing of the 16S rRNA V3-V4 region as described by Lei et al. [15]. Specifically, polymerase chain reaction (**PCR**) amplification was performed in triplicate using primers including 338F (forward: 5′-ACTCCTACGGGAGGCAGCAG-3′) and 806R (reverse: 5′-GGACTACHVGGGTWTCTAAT -3′), which were labeled by a unique barcode to distinguish different samples [16]. Then PCR products from the same bacterial DNA sample were combined, purified, and quantified. These were used for Miseq library preparation and paired-end sequencing on the Illumina Miseq platform.

### 2.3. Data Processing and Statistical Analysis

Raw sequencing reads from 16S rRNA sequencing were screened to remove low-quality data, merged based on overlaps, and separated into different samples according to the unique barcode. The clean reads were obtained by removing barcodes, primers, and chimeras. Operational taxonomic units (**OTUs**) above 97% similarity were clustered using QIIME version 1.8, and they were then used for microbiota diversity and taxonomic analysis. The α diversity of microbiota for each treatment was evaluated by Chao 1 and Shannon indices to assess microbial richness and evenness. The difference in growth performance, intestinal index, and α diversity of microbiota was analyzed using two-way ANOVA and Tukey’s test. The β diversity of the microbiota community between treatments was assessed using Principal coordinates analysis (**PCoA**) and Adonis analysis based on Bray–Curtis distance. Adonis analysis was used to determine the main effects of breed and nutrient density, as well as their interaction effects on microbiota community composition. Pairwise comparisons were performed following Adonis analysis to assess significant differences between treatments. Multivariate homogeneity of group dispersion was determined prior to Adonis analysis to ensure that the observed differences between breeds or diets were due to differences in centroids, rather than differences in the dispersion of samples within groups. OTUs were annotated by comparing their sequence with the Silva 138 database using the Basic Local Alignment Search Tool (BLAST 2.12.0) [15]. Differential genera in the ileum and cecum of chickens were identified through the Scheirer–Ray–Hare test on d7, 21, and 42. Dunn’s test was applied when significant interactions between breed and dietary nutrient density were observed. Significance was set at less than 0.05. Spearman correlations between relative abundance of bacteria genera and phenotype parameters were analyzed separately for the AA and BY chickens, due to significant differences in phenotype parameters between breeds. Significantly strong correlation was considered as 0.6 ≤ |R| < 0.80, and moderate correlation was considered as 0.4 ≤ |R| < 0.60, when *p* was less than 0.05 [17]. If the 0.80 ≤ |R| < 1.0, and *p* < 0.05, the correlation was considered very strong. Genera with an average abundance of less than 0.01% were excluded from the differential genera screening and Spearman correlation analysis to minimize the type I error [18].

## 3. Results

### 3.1. Growth Performance

Table 2 presents the effects of breed and dietary nutrient density on chicken growth performance. Significant interactions between breed and nutrient density were observed for ADG, body weight (**BW**), average daily feed intake (**ADFI**), feed intake (**FI**), and FCR. Throughout the study period (d0 to d42), the LN diet reduced ADG and BW (d42) in AA broilers but had no impact on BY chickens. Conversely, the LN diet increased ADFI, FI, and FCR in BY chickens while not affecting AA broilers. The interaction between breed and diet was most pronounced during the early stages (d0 to d22). Specifically, from d0 to d7 and d8 to d22, the LN diet decreased ADG in AA broilers but did not affect BY chickens. In the later stages (d22 to d42), the main effect of dietary nutrient density became more apparent. The LN diet led to a decrease in ADG and an increase in ADFI compared to the HN diet. Additionally, the LN diet increased FCR from d8 to d21 and d22 to d42. Overall, AA broilers consistently exhibited higher ADG and ADFI and a lower FCR compared to BY chickens throughout the study period. However, the mortality of AA broilers was greater than BY chickens.

### 3.2. Intestinal Length and Weight

Interactions between breed and diet were observed for intestinal length and weight during the earlier stages (Table 3 and Table 4). In AA broilers, the LN diet reduced the jejunal absolute length on d7 and decreased the absolute weight of the jejunum and ileum on d7 and d21, as well as the duodenum on d21. These effects were not observed in BY chickens. Dietary nutrient density significantly affected duodenal absolute length on d21 and weight on d7, with both measurements being lower under the LN diet compared to the HN diet. However, dietary nutrient density did not significantly alter the length and weight of the duodenum, jejunum, or ileum in the later stage (d42), nor did it affect cecal measurements throughout the study period. Additionally, the absolute length and weight of the small intestine (duodenum, jejunum, and ileum) and cecum were generally greater in AA broilers compared to BY chickens on d7, d21, and d42.

### 3.3. Microbial Diversity

**Figure 1:**
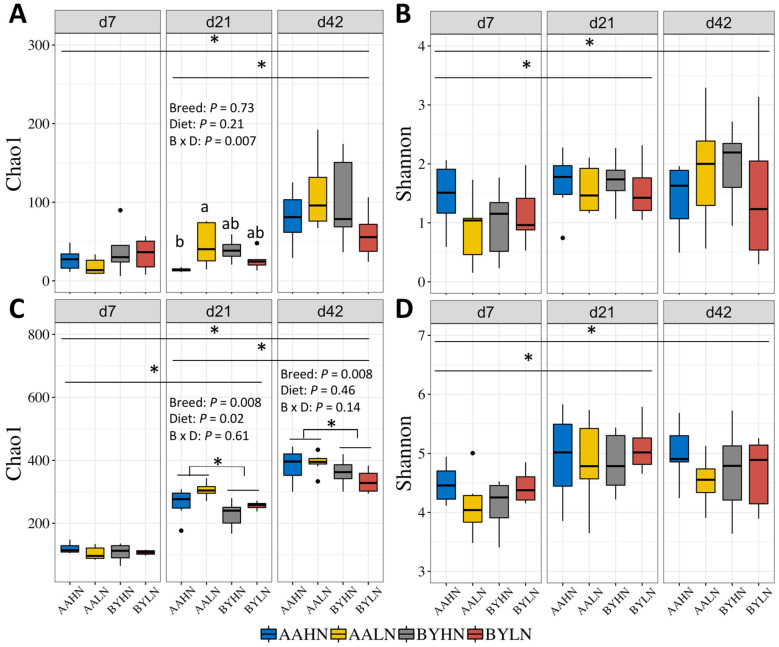
Alpha diversity for bacteria in the ileum and cecum of chickens: (**A**): Chao1 index of microbial community in the ileum. (**B**): Shannon index of microbial community in the ileum. (**C**): Chao1 index of microbial community in the cecum. (**D**): Shannon index of microbial community in the cecum. AAHN: Arbor Acres broilers fed a high-nutrient diet; AALN: Arbor Acres broilers fed a low-nutrient diet; BYHN: Beijing-You chickens fed a high-nutrient diet; BYLN: Beijing-You chickens fed a low-nutrient diet. The experiment unit was chicken. The difference between treatments were analyzed using two-way ANOVA and Tukey’s test. Asterisks indicate significant difference between two time points or breeds. Significance was set as *p* value less than 0.05. Black dots outside the whisker range of boxplots represent outliers.

Interactions between dietary nutrient density and breed affected the Chao1 index. Specifically, the LN diet increased the Chao1 index in the ileum of AA broilers on d21 but had no effect in BY chickens (Figure 1A). Dietary nutrient density also significantly influenced the Chao1 index in the cecum on d21, with higher values in the LN groups. Breed significantly affected the Chao1 index in the cecum, showing more in AA broilers compared to BY chickens on d21 and d42. Additionally, the Chao1 index increased over time from d21 to d42, and in the cecum from d7 to d21 and from d21 to d42 (Figure 1A,C). Similar trends were observed in the Shannon index in both the ileum (Figure 1B) and cecum (Figure 1D). However, breed and dietary nutrient density did not influence the Shannon index at any developmental stage.

**Figure 2:**
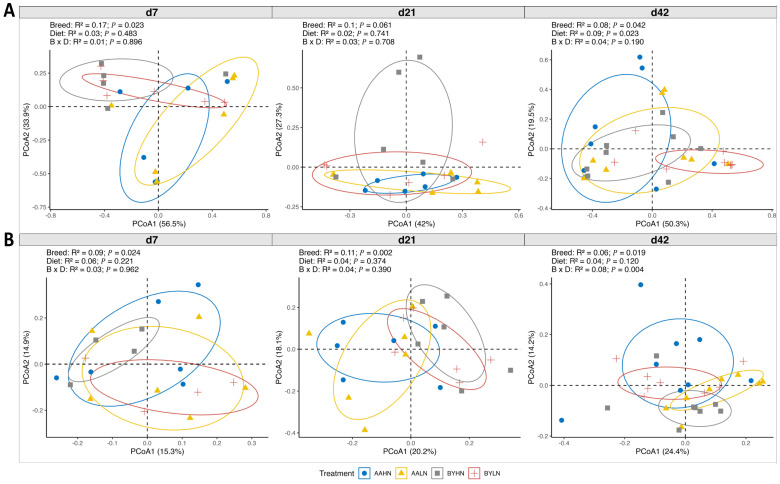
Beta diversity for microbiota in the ileum (**A**) and cecum (**B**) of chickens. AAHN: Arbor Acres broilers fed a high-nutrient diet; AALN: Arbor Acres broilers fed a low-nutrient diet; BYHN: Beijing-You chickens fed a high-nutrient diet; BYLN: Beijing-You chickens fed a low-nutrient diet. The experiment unit was chicken. The diversity of microbiota community composition between breeds was determined by Principal coordinates analysis (PCoA) and Adonis analysis using Bray–Curtis distance. Significance was set as *p* value less than 0.05.

The β diversity analysis showed differences in microbiota community composition between breeds in the ileum and cecum from d7 to d42 (*p* < 0.05), except in the ileum on d21 (0.05 < *p* < 0.1, Figure 2). Dietary nutrient density affected microbial composition in the ileum only on d42. A significant interaction between breed and nutrient density was observed for the cecal microbiota on d42 (Figure 2B). Pairwise comparisons revealed differences in microbial composition between breeds and dietary nutrient densities on d42 (Table S1). Multivariate homogeneity tests showed no difference in group dispersion homogeneity.

### 3.4. Microbial Composition at the Genus Level

**Figure 3:**
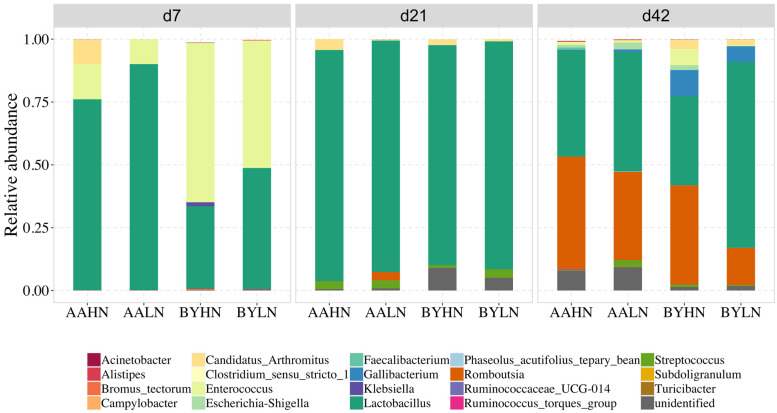
Relative abundance of the top 20 genera in the ileum of chickens. AAHN: Arbor Acres broilers fed a high-nutrient diet; AALN: Arbor Acres broilers fed a low-nutrient diet; BYHN: Beijing-You chickens fed a high-nutrient diet; BYLN: Beijing-You chickens fed a low-nutrient diet.

Distinct differences in predominant genera were observed between AA and BY chickens in the ileum on d7 (Figure 3). In AA broilers, *Lactobacillus* dominated the bacterial composition, accounting for 75–90%, while in BY chickens, it represented only 32–48%. Conversely, *Enterococcus* constituted about 10% of the ileal microbiota in AA broilers but 51–63% in BY chickens on d7. By d21, *Lactobacillus* became the most predominant genus in both breeds, comprising approximately 90% of the ileal microbiota. By d42, the ileal microbiota shifted, with *Lactobacillus* comprising 35–75% and *Romboutsia* 15–45% in both AA and BY chickens.

**Figure 4:**
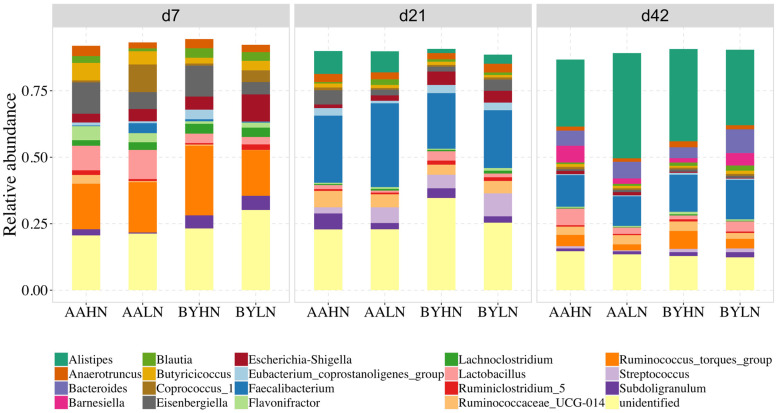
Relative abundance of the top 20 genera in the cecum of chickens. AAHN: Arbor Acres broilers fed a high-nutrient diet; AALN: Arbor Acres broilers fed a low-nutrient diet; BYHN: Beijing-You chickens fed a high-nutrient diet; BYLN: Beijing-You chickens fed a low-nutrient diet.

The cecal bacterial composition was more diverse compared to the ileum, with the top 5 genera accounting for over 65% in the cecum and 95% in the ileum (Figure 4). On d7, *Ruminococcus_torques_group* was most predominant in the cecum (~20%), followed by *Eisenbergiella* (9%) and *Lactobacillus* (7%). By d21, *Faecalibacterium* dominated the cecum, constituting around 25% of the total genera abundance. However, by d42, *Alistipes* became the most enriched genus in the cecum at 32%, followed by *Faecalibacterium* at 13%.

### 3.5. Differential Bacteria at the Genus Level

**Figure 5:**
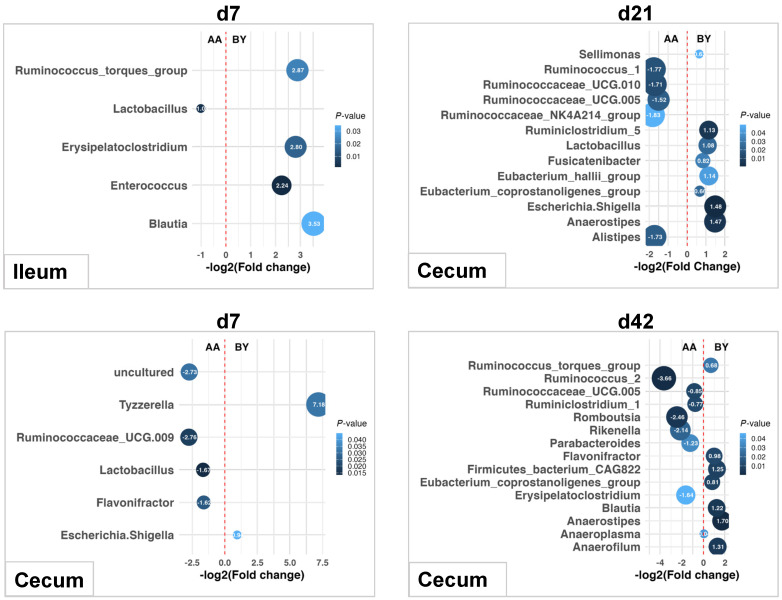
Differential bacterial genera in the ileum and cecum of two distinct chicken breeds. AA: Arbor Acres broilers; BY: Beijing-You chickens. Differences in the relative abundance of genera were analyzed using R software (RStudio 1.1.463). Significance was set at less than 0.05.

The Scheirer–Ray–Hare test detected the significant main effects of breed and dietary nutrient density, as well as their interactions on the relative abundances of bacterial genera. The main effects of breed on the relative abundance of genera were observed in the ileum on d7 and cecum on d7, d21, and d42 (Figure 5). On d7, *Lactobacillus* was more abundant in the ileum of AA broilers, while BY chickens had higher abundances of *Ruminococcus_torques_group*, *Erysipelatoclostridium*, *Enterococcus*, and *Blautia* in the ileum. In the cecum, AA broilers exhibited higher levels of *Ruminococcaceae_UCG.009*, *Lactobacillus*, and *Flavonifractor*, whereas BY chickens had more *Tyzzerella* and *Escherichia.Shigella*. On d21, AA broilers were enriched with *Ruminococcus_1*, *Ruminococcaceae_UCG.010*, *Ruminococcaceae_UCG.005*, *Ruminococcaceae_NK4A214_group*, and *Alistipes* in the cecum, while BY chickens had higher abundances of *Sellimonas*, *Ruminiclostridium_5*, *Lactobacillus*, *Fusicatenibacter*, *Eubacterium_hallii_group*, *Eubacterium_coprostanoligenes_group*, *Escherichia.Shigella*, and *Anaerostipes* in the cecum. By d42, AA broilers had seven enriched genera in the cecum, including *Ruminococcaceae_UCG.005*, *Romboutsia*, *Parabacteroides*, and *Erysipelatoclostridium*, while BY chickens had eight more abundant genera, including *Ruminococcus_torques_group*, *Flavonifractor*, *Eubacterium_coprostanoligenes_group*, *Blautia*, *Anaerostipes*, and *Anaerofilum* in the cecum.

**Figure 6:**
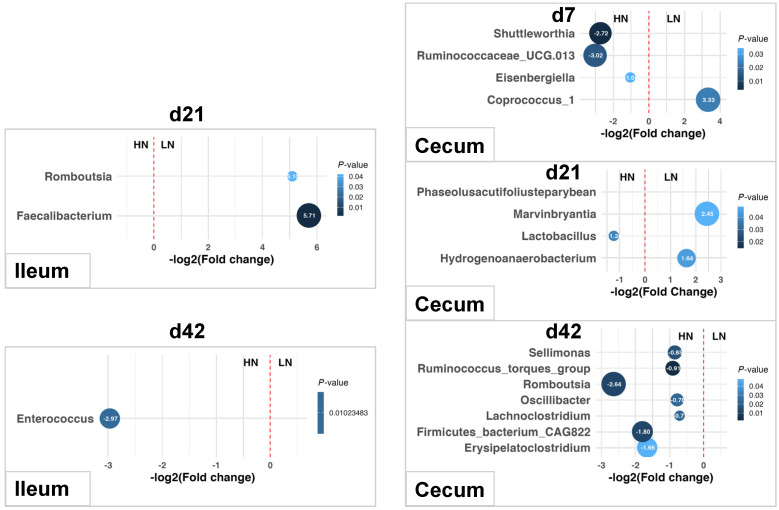
Differential bacterial genera in the ileum and cecum of chicken on different nutrient density diets. HN: a high-nutrient diet; LN: a low-nutrient diet. Differences in genera were analyzed with the Scheirer–Ray–Hare test using R software (RStudio 1.1.463). Significance was set at less than 0.05.

Dietary nutrient density also influenced the relative abundances of genera in the ileum and cecum of chickens (Figure 6). *Romboutsia* and *Faecalibacterium* were enriched in the ileum of chickens fed the LN diet on d21, while *Enterococcus* was more abundant in the ileum of chickens fed the HN diet on d42. In the cecum, chickens on the HN diet had higher levels of *Shuttleworthia*, *Ruminococcaceae_UCG.013*, and *Eisenbergiella* on d7, whereas those on the LN diet were enriched in *Coprococcus_1* on d7. By d21, the HN diet increased *Lactobacillus* abundance in the cecum, while the LN diet led to higher levels of *Hydrogenoanaerobacterium* and *Marvinbryantia* in the cecum. On d42, the HN diet resulted in increased relative abundances of *Sellimonas*, *Ruminococcus_torques_group*, *Romboutsia*, *Oscillibacter*, *Lachnoclostridium*, and *Erysipelatoclostridium* in the cecum.

**Figure 7:**
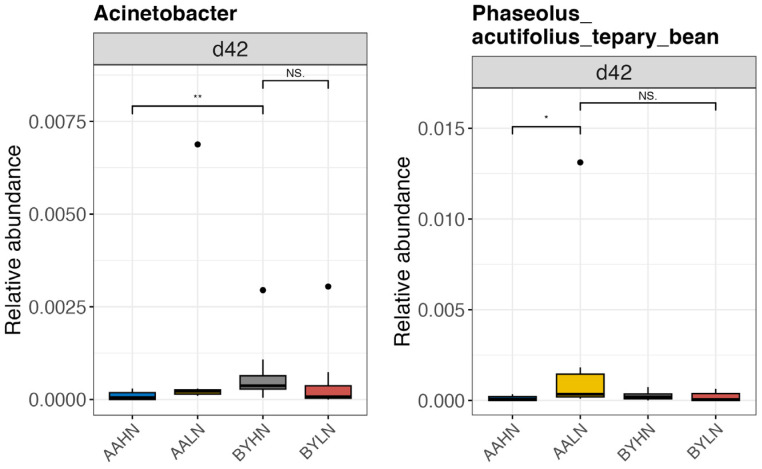
Differential bacterial genera in the ileum of chickens on day 42. AAHN: Arbor Acres broilers fed a high-nutrient diet; AALN: Arbor Acres broilers fed a low-nutrient diet; BYHN: Beijing-You chickens fed a high-nutrient diet; BYLN: Beijing-You chickens fed a low-nutrient diet. Differences in genera were analyzed using the Scheirer–Ray–Hare test and Dunn’s test using R software (RStudio 1.1.463). Significance was set at less than 0.05. Asterisks indicate significant difference between the two treatment groups. NS. indicates there is no statistically significant difference between groups. Black dots outside the whisker range of boxplots represent outliers.

**Figure 8:**
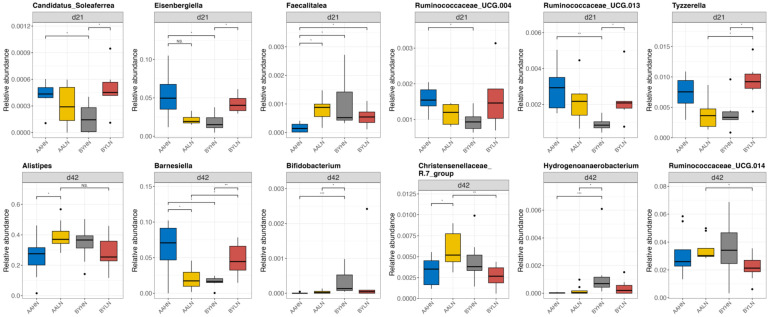
Differential bacterial genera in the cecum of chickens on day 21 and day 42. AAHN: Arbor Acres broilers fed a high-nutrient diet; AALN: Arbor Acres broilers fed a low-nutrient diet; BYHN: Beijing-You chickens fed a high-nutrient diet; BYLN: Beijing-You chickens fed a low-nutrient diet. Differences in genera were analyzed using the Scheirer–Ray–Hare test and Dunn’s test using R software (RStudio 1.1.463). Significance was set at less than 0.05. Asterisks indicate significant difference between the two treatment groups. NS. indicates there is no statistically significant difference between groups. Black dots outside the whisker range of boxplots represent outliers.

Significant interactions between breed and diet were observed in the ileum for two genera (Figure 7) and in the cecum for twelve genera (Figure 8). In AA broilers, the LN diet led to an increased relative abundance of *Phaseolus_acutifolius_tepary_bean* in the ileum on d42, *Faecalitalea* in the cecum on d21, and *Alistipes* and *Christensenellaceae_R.7_group* in the cecum on d42. Conversely, in BY chickens, the LN diet increased the relative abundance of *Candidatus_Soleaferrea*, *Eisenbergiella*, *Ruminococcaceae_UCG.013*, and *Tyzzerella* in the cecum on d21. Additionally, *Barnesiella* abundance decreased in the cecum of AA broilers on the LN diet, but increased in BY chickens on d42.

**Figure 9:**
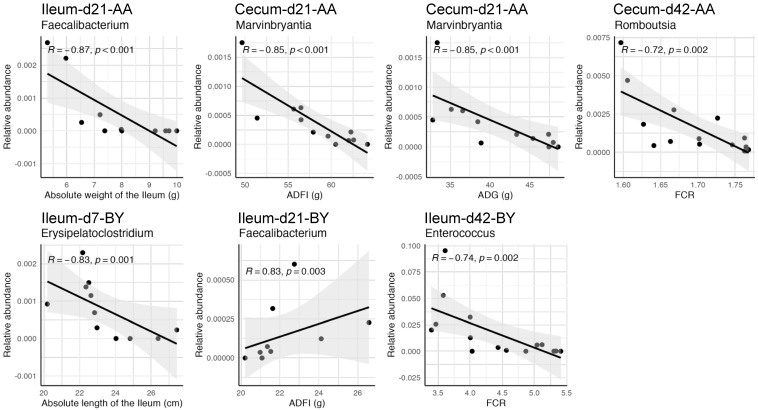
Significant correlations between differential genera and phenotypic traits of chickens. AA: Arbor Acres broilers. BY: Beijing-You chickens. The X-axis represents the relative abundance of genera on d7, d21, and d42. The Y-axis represents growth parameters, including body weight (BW), and absolute length and weight of the ileum at those respective time points, along with average daily gain (ADG), average daily feed intake (ADFI), and feed conversion ratio (FCR) from d0 to d7, from d8 to d21, and from d22 to d42.

Scatter plots showed significant correlations between differential genera and phenotypic traits across intestinal segments and time points (Figure 9). On d7, *Erysipelatoclostridium* in the ileum had a very strong negative correlation with ileum length in BY chickens (0.80 ≤ |R| < 1.0). On d21, *Faecalibacterium* in the ileum showed a very strong negative correlation with ileum weight in AA broilers, and a very strong positive correlation with ADFI in BY chickens. *Marvinbryantia* in the cecum was very strongly negatively correlated with ADFI and ADG in AA broilers. On d42, *Enterococcus* in the ileum and *Romboutsia* in the cecum were strongly negatively correlated with FCR in BY chickens and AA broilers, respectively (0.6 ≤ |R| < 0.80).

## 4. Discussion

This study revealed differences in growth performance, intestinal measurements, and gut microbiota composition between two chicken breeds, AA and BY, at various growth stages. Nutrient density influenced growth performance and microbiota composition in the ileum and cecum, with notable interactions between breed and dietary nutrient density.

### 4.1. Effects of Breed

#### 4.1.1. Growth Performance

AA broilers showed a greater growth performance compared to BY chickens, with lower FCR and ADFI, and higher ADG, which was mainly due to genetic selection. Zhao et al. [7] and Havenstein et al. [19] also reported dramatic differences in growth performance between commercial broilers and local breeds. Furthermore, AA broilers had greater intestinal length and weight throughout the study, aligning with Lumpkins et al. [20], who reported that modern chicken breeds had longer villi in the jejunum and ileum compared to a historic strain Athens Canadian Random Bred across all experimental periods. These findings suggest that fast-growing chickens have enhanced intestinal development to support better nutrient digestion and absorption.

#### 4.1.2. Microbiota Composition

The microbial communities in both the ileum and cecum were consistently differentiated by breed. AA broiler had a higher abundance of *Lactobacillus* in both the ileum and cecum, along with members of the *Ruminococcaceae* family in the cecum including *Ruminococcus_1*, *Ruminococcus_2*, *Ruminococcaceae_UCG.005*, *Ruminococcaceae_UCG.009*, *Ruminococcaceae_UCG.010*, and *Ruminococcaceae_NK4A214_group*. *Lactobacillus*, known for its probiotic properties, ferments carbohydrates into lactic acid, promoting intestinal health and growth performance [21]. The *Ruminococcaceae* family, which degrades indigestible polysaccharides and produces SCFA, also contributes to gut health and growth performance [22,23,24,25]. These suggest that genetic selection in AA broilers has favored a microbiota composition that provides additional energy and enhances growth performance [18].

BY chickens showed higher levels of genera involved in fiber fermentation and SCFA production, such as *Ruminococcus_torques_group* and *Blautia*, which have been associated with increased body weight in chickens [26,27]. Additionally, BY chickens had higher levels of lactic acid-producing *Enterococcus* and butyrate-producing *Tyzzerella* on d7, along with greater *Anaerostipes* abundance in later stages [28,29,30,31]. Although these SCFA-producing bacteria could potentially improve gut health, they did not fully compensate for the shorter intestine and lower growth performance observed in BY chickens. *Erysipelatoclostridium*, an opportunistic pathogen, was enriched in the ileum of BY chickens on d7 but shifted to the cecum of AA broilers by d42 [32]. Conversely, beneficial *Lactobacillus* and *Flavonifractor*, producing acetic acid and butyric acid, were enriched in AA broilers on d7, but were more dominant in BY chickens in later stages on d21 and d42 [33]. The shifts in bacterial genera between breeds indicate that AA broilers developed a more beneficial microbiota early on, whereas BY chickens exhibited a more favorable microbiota later, potentially enhancing pathogen resistance and intestinal health.

### 4.2. Effect of Nutrient Density

#### 4.2.1. Growth Performance

Low dietary nutrient density led to reduced ADG in AA broilers during early development stages, but did not impact BY chickens in the same way. This might be due to impaired intestinal development in AA broilers under low-nutrient conditions [34,35]. Rapid development of the small intestine occurs during the early stages, with significant growth between d5 and d7 [36]. The impaired small intestine development due to reduced nutrient density could hinder growth performance in AA broilers. In contrast, BY chickens compensated for reduced nutrient density by increasing feed intake, suggesting greater adaptability to nutrient reductions, as previously reported by Zhao et al. [7]. This adaptability in BY chickens may be due to their lower growth rate and nutrient demands, as previous studies have also shown that slow-growing chickens are more resilient to changes in diet [7,37]. However, there was a main effect of nutrient density in the later stage, with decreased ADG and increased ADFI in the LN group, likely driven by the heightened nutrient demands associated with rapid growth rate.

#### 4.2.2. Microbiota Composition

Low-nutrient diets increased α diversity of the microbiota in the cecum on d21, contrary to other studies that reported no change or even decreased diversity [11,12,13]. This increase might be attributed to the diet modification involved in corn, which contains around 10% non-starch polysaccharides fermentable by beneficial bacteria [38,39,40,41]. In the ileum, only AA broilers showed increased α diversity under low nutrient density, indicating a higher susceptibility compared to BY chickens. Nutrient density also affects the β diversity of microbial composition in both the ileum and cecum on d42, likely due to the prolonged effects of dietary nutrient density.

During the early growth stage on d7, the LN group significantly reduced the abundance of *Shuttleworthia*, *Eisenbergiella*, and *Ruminococcaceae UCG-013* in the cecum. Both *Shuttleworthia* and *Eisenbergiella* are associated with anti-inflammatory effects [42,43], so their reduction may have weakened the anti-inflammatory defenses in the LN group. The decline in *Ruminococcaceae UCG-013* and *Eisenbergiella* is linked to *Salmonella* and *Eimeria tenella* infections, further suggesting a negative impact on cecal health in the LN group during this early stage [44,45].

In the middle-growth phase on d21, the LN diet increased the abundance of *Faecalibacterium* and *Romboutsia* in the ileum. Previous studies have shown that low-fat, high-fiber diets can increase *Faecalibacterium* abundance and reduce inflammatory markers [46]. *Romboutsia* increased in the ileum of broilers supplemented with probiotic cocktails and positively correlated with chicken secretory immunoglobulin A levels [47]. The enrichment of anti-inflammatory and immune-regulating bacteria like *Faecalibacterium* and *Romboutsia* in the LN groups suggests that reduced nutrient density might benefit intestinal health [48,49]. However, in this study, *Faecalibacterium* abundance in the ileum was negatively correlated with the ileal absolute weight of AA broilers and positively correlated with the ADFI in BY chickens. This indicates that the increased *Faecalibacterium* abundance in the ileum, driven by reduced nutrient density, has different effects on these distinct breeds, potentially impairing intestinal development in AA broilers, while benefiting adaptation to nutrient reduction in BY chickens. In the cecum, the LN diet generally increased *Hydrogenoanaerobacterium*, a bacterium positively correlated with body weight gain [50]. However, LN also increased proinflammatory bacteria *Marvinbryantia* [51], which was negatively correlated with ADFI and ADG in AA broilers. Additionally, LN reduced beneficial bacteria such as *Lactobacillus* on d21. These changes may have contributed to the impaired growth performance observed in the LN chickens. And the increased *Marvinbryantia* might reduce the adaptation of AA broilers to reduced nutrient density.

In the late growth stage on d42, the LN groups significantly decreased the abundance of *Enterococcus* in the ileum, which was negatively correlated with FCR in BY chickens. *Enterococcus*, known to serve as a dietary probiotic that promotes intestinal integrity and growth performance [29,52,53], may be associated with decreased feed efficiency in BY chickens due to its reduced abundance in the LN group. In the cecum, the LN group exhibited lower relative abundances of *Ruminococcus_torques_group* and *Romboutsia*, consistent with the previous studies [54,55]. *Ruminococcus_torques*, a mucin degrader, can potentially compromise the intestinal barrier through enzymatic activity [56,57]. Several operational taxonomic units (**OTUs**) of *Romboutsia* were associated with increased intestinal permeability [58]. Thus, the reduced abundance of these two genera in the LN group may have helped to maintain the integrity of the intestinal mucin barrier. Furthermore, *Ruminococcus_torques_group* and *Romboutsia* abundances were positively correlated with hepatic triglyceride content and fat deposition in poultry [55,59]. Therefore, the decrease in these genera in the LN group may have contributed to reduced fat deposition and improved lipid metabolism. Similarly, *Erysipelatoclostridium* showed a lower abundance in the LN group, consistent with previous studies linking increased *Erysipelatoclostridium* abundance to higher fat deposition, lipid metabolites, BW, and ADG [11,12,50,60,61]. Additionally, *Oscillibacter* reduction in the LN groups has been associated with lower residual feed intake and higher abdominal fat deposition [18,62]. These changes suggest that reduced nutrient density may have promoted the integrity of the intestinal barrier and regulated lipid metabolism through microbiota modulation.

### 4.3. The Interactive Effects of Nutrient Density and Breed on Microbiota

On d21, the LN diet significantly increased the abundance of *Ruminococcaceae_UCG.013*, *Eisenbergiella*, and *Tyzzerella* in the cecum of BY chickens. *Ruminococcaceae_UCG.013* and *Eisenbergiella* are involved in the degradation of indigestible carbohydrates such as cellulose and hemicellulose, producing short-chain fatty acids and contributing to obesity resistance [63,64]. Notably, both *Ruminococcaceae_UCG.013* and *Eisenbergiella* typically decrease following *Salmonella Typhimurium* and *Eimeria tenella* infection [44,45]. Additionally, *Tyzzerella* abundance has been reported to decrease under heat stress and subclinical necrotic enteritis challenges but can be enriched by probiotic supplementation with *Bacillus licheniformis* H2 [14,65]. These findings suggest that the LN diet may positively influence the gut health of BY chickens during the middle-growth phase by regulating the microbiota. In AA broilers, although the LN diet did not significantly affect *Tyzzerella* abundance, a previous study showed a decreased *Tyzzerella* abundance in a reduced ME diet (50 kcal/kg lower) [11]. This indicates that the effect of reduced nutrient density on *Tyzzerella* abundance might differ between fast- and slow-growing chicken breeds, potentially affecting gut health. In this study, the LN diet enriched *Faecalitalea* in the cecum of AA broilers. *Faecalitalea* produces lactic acid and butyrate, which play roles in anti-inflammation and intestinal barrier protection [66,67,68]. Thus, the increase in *Faecalitalea* due to the LN diet may positively influence intestinal health in AA broilers during the mid-growth phase.

On d42, the LN diet increased the abundance of *Christensenellaceae_R-7_group* and *Alistipes* in the cecum of AA broilers. *Christensenellaceae_R-7_group* has been positively associated with the expression of tight junction proteins in both chickens and piglets, suggesting a potential role in maintaining intestinal barrier integrity under nutrient-restricted conditions [42,69]. *Alistipes*, a propionate producer, was negatively correlated with pro-inflammatory cytokines in broilers [70,71]. However, *Alistipes* has also been associated with necrotic enteritis and *Eimeria tenella* challenges [42,72]. Similarly, *Barnesiella*, which increased in BY chickens fed the LN diet, is known for producing butyric- and iso-butyric acid with anti-inflammatory properties that can prevent pathogen overgrowth [73,74]. However, *Barnesiella* has also been linked to necrotic enteritis and Campylobacter colonization [42,75]. These findings suggest that the LN diet may enhance the host immune response to potential pathogen infection in both slow-growing BY chickens and fast-growing AA broilers during the late-growth phase. Interestingly, *Barnesiella* abundance decreased in AA broilers fed the LN diet.

## 5. Conclusions

Both breed and dietary density have significant impacts on chicken growth performance and gut microbiota composition. AA broilers, due to genetic selection, typically exhibit better growth performance and intestinal development. In contrast, BY chickens demonstrate greater adaptability under low nutrient density. The impact of an LN diet on gut microbiota varies over time, potentially leading to different growth performance and gut health states. The interaction effects between breed and diet further influence the composition, indicating that practical feeding strategies should consider both breed characteristics and nutritional strategies to optimize production performance and health.

## Figures and Tables

**Table 1:** Feed ingredients and nutrient composition of high- and low-nutrient diets.

Feed Ingredients (%)	HN ^1^	LN ^2^	Nutrient Composition	HN ^1^	LN ^2^
Corn	51.73	60.05	Metabolizable energy (Kcal/Kg)	3053	2900
Soybean meal	34.71	32.91	Crude protein (%)	23	21
Corn gluten meal	5.1	2.72	Lysine (%)	1.34	1.05
Soybean oil	4.3	0.89	Methionine (%)	0.53	0.40
Calcium hydrogen phosphate	1.9	1.88	Calcium (%)	0.96	0.96
Limestone	0.75	0.78	Available phosphorus (%)	0.45	0.45
NaCl	0.3	0.3			
Trace mineral mixture ^3^	0.2	0.2			
Choline chloride	0.5	0.16			
L-Lysine hydrochloride	0.3	——			
DL-Methionine	0.17	0.07			
Vitamins ^4^	0.02	0.02			
Ethoxyquin	0.02	0.02			
Total	100	100			

^1^ HN refers to a high-nutrient diet; ^2^ LN refers to a low-nutrient diet. ^3^ The trace mineral mixture consists of the following (per kilogram of diet): copper, 8 mg (CuSO_4_·5H_2_O); zinc, 100 mg (ZnSO_4_·H_2_O); iron, 80 mg (FeSO_4_); manganese, 100 mg (MnSO_4_·H_2_O); iodine, 0.35 mg (KI); selenium, 0.15 mg (Na_2_SeO_3_). ^4^ Vitamins consist of the following (per kilogram of diet): vitamin A, 9500 IU; vitamin D_3_, 3000 IU; vitamin E, 30 IU; vitamin K_3_, 3 mg; vitamin B_1_, 2 mg; vitamin B_2_, 6 mg; vitamin B_12_, 0.025 mg; niacin, 50 mg; pantothenic acid, 12 mg; folic acid, 1.25 mg; biotin, 0.0325 mg.

**Table 2:** Effect of breed and dietary nutrient density on the growth performance in chickens.

Items	Treatment Groups				SEM	*p*-Values		
	AAHN	AALN	BYHN	BYLN		Breed	Diet	B × D
ADG, g BW/d								
0–7 d	13.6 ^a^	10.9 ^b^	2.1 ^c^	2.3 ^c^	0.26	<0.001	<0.001	<0.001
8–21 d	46.8 ^a^	36.6 ^b^	11.3 ^c^	10.5 ^c^	0.57	<0.001	<0.001	<0.001
22–42 d	82.2	79.4	15.0	14.7	0.71	<0.001	0.029	0.085
0–42 d	58.9 ^a^	53.9 ^b^	11.6 ^c^	11.1 ^c^	0.46	<0.001	<0.001	<0.001
ADFI, g feed/d								
0–7 d	25.7	25.2	10.0	11.0	0.41	<0.001	0.534	0.053
8–21 d	59.7 ^a^	55.5 ^a^	21.3 ^b^	23.1 ^b^	1.18	<0.001	0.330	0.018
22–42 d	135.6	138.2	59.9	68.7	1.59	<0.001	0.001	0.058
0–42 d	94.6 ^a^	94.9 ^a^	39.4 ^c^	44.7 ^b^	1.16	<0.001	0.022	0.038
FCR, g feed/g BW								
0–7 d	1.90	2.32	4.89	4.84	0.257	<0.001	0.484	0.371
8–21 d	1.27	1.52	1.89	2.20	0.034	<0.001	<0.001	0.336
22–42 d	1.65	1.74	4.06	4.72	0.163	<0.001	0.030	0.097
0–42 d	1.60 ^c^	1.76 ^c^	3.43 ^b^	4.02 ^a^	0.087	<0.001	<0.001	0.019
FI, kg								
0–42 d	4.0 ^a^	4.0 ^a^	1.7 ^b^	1.9 ^c^	0.05	<0.001	0.022	0.038
BW, kg								
d42	2.5 ^a^	2.3 ^b^	0.5 ^c^	0.5 ^c^	0.02	<0.001	<0.001	<0.001
Mortality, %								
0–42 d	1.92	4.81	0.00	0.00	0.944	0.001	0.138	0.138

AALN: Arbor Acres broilers fed a low-nutrient diet; BYHN: Beijing-You chickens fed a high-nutrient diet; BYLN: Beijing-You chickens fed a low-nutrient diet. FI: feed intake; BW: body weight. The experiment unit was a cage (*n* = 8). The difference between treatments within age were analyzed using two-way ANOVA and Tukey’s test. Different letters indicate significant difference (*p* < 0.05) between treatment groups in a certain growth phase.

**Table 3:** Effect of breed and dietary nutrient density on the absolute intestinal length.

Items	Treatment Groups				SEM	*p*-Values		
	AAHN	AALN	BYHN	BYLN		Breed	Diet	B × D
Duodenum (cm)								
d7	18.9	18.6	14.1	15.5	0.50	<0.001	0.289	0.099
d21	25.4	22.8	17.1	17.1	0.62	<0.001	0.045	0.055
d42	32.5	30.3	21.8	21.9	0.8	<0.001	0.193	0.136
Jejunum (cm)								
d7	41.0 ^a^	32.8 ^b^	27.8 ^b^	29.2 ^b^	1.77	<0.001	0.067	0.012
d21	58.9	54.2	36.7	35.7	1.54	<0.001	0.074	0.236
d42	74.2	78.2	43.4	46.7	2.15	<0.001	0.099	0.875
Ileum (cm)								
d7	39.2 ^a^	35.0 ^a^	22.5 ^b^	24.0 ^b^	1.36	<0.001	0.333	0.044
d21	56.7	56.9	34.2	34.5	1.28	<0.001	0.839	0.946
d42	77.9	79.8	40.7	42.7	2.02	<0.001	0.361	0.980
Cecum (cm)								
d7	12.1	12.3	9.3	10.7	0.59	0.001	0.210	0.306
d21	22.3	21.4	14.8	15.7	0.69	<0.001	0.955	0.229
d42	40.9	39.4	22.7	23.0	0.81	<0.001	0.458	0.274

AAHN: Arbor Acres broilers fed a high-nutrient diet; AALN: Arbor Acres broilers fed a low-nutrient diet; BYHN: Beijing-You chickens fed a high-nutrient diet; BYLN: Beijing-You chickens fed a low-nutrient diet. The experiment unit was chicken (*n* = 8). The difference between treatments within age were analyzed using two-way ANOVA and Tukey’s test. Different letters indicate significant difference (*p* < 0.05) between treatment groups in a certain growth phase.

**Table 4:** Effect of breed and dietary nutrient density on the absolute intestinal weight.

Items	Treatment Groups				SEM	*p*-Values		
	AAHN	AALN	BYHN	BYLN		Breed	Diet	B × D
Duodenum (g)								
d7	3.3	2.6	1.3	1.2	0.18	<0.001	0.038	0.108
d21	4.9 ^a^	3.4 ^b^	1.6 ^c^	1.5 ^c^	0.19	<0.001	<0.001	0.001
d42	15.9	13.7	5.5	5.6	0.69	<0.001	0.135	0.103
Jejunum (g)								
d7	4.8 ^a^	3.7 ^b^	1.8 ^c^	1.7 ^c^	0.24	<0.001	0.010	0.043
d21	13.0 ^a^	9.2 ^b^	3.7 ^c^	3.3 ^c^	0.41	<0.001	<0.001	<0.001
d42	26.5	24.5	7.3	7.2	0.71	<0.001	0.132	0.203
Ileum (g)								
d7	3.2 ^a^	2.4 ^b^	1.1 ^c^	1.2 ^c^	0.15	<0.001	0.022	0.007
d21	8.8 ^a^	6.8 ^b^	2.3 ^c^	2.4 ^c^	0.34	<0.001	0.009	0.005
d42	20.7	17.8	4.8	4.8	0.74	<0.001	0.065	0.064
Cecum (g)								
d7	0.9	0.8	0.4	0.4	0.04	<0.001	0.570	0.167
d21	2.7	2.4	1.0	1.2	0.13	<0.001	0.753	0.108
d42	7.4	6.6	2.6	2.5	0.26	<0.001	0.094	0.218

AAHN: Arbor Acres broilers fed a high-nutrient diet; AALN: Arbor Acres broilers fed a low-nutrient diet; BYHN: Beijing-You chickens fed a high-nutrient diet; BYLN: Beijing-You chickens fed a low-nutrient diet. The experiment unit was chicken (*n* = 8). The difference between treatments within age were analyzed using two-way ANOVA and Tukey’s test. Different letters indicate significant difference (*p* < 0.05) between treatment groups in a certain growth phase.

## Data Availability

The datasets supporting this article are available from the corresponding author on reasonable request.

## Supplementary Materials

The following supporting information can be downloaded at: https://www.mdpi.com/article/10.3390/ani14233528/s1, Table S1: Pairwise comparison between treatments for beta diversity of microbiome in the cecum of chickens on d42.

## List of Abbreviations

AA	Arbor Acres
BY	Beijing-You
HN	High-nutrient
LN	Low-nutrient
AAHN	AA broilers fed the HN diet
AALN	AA broilers fed the LN diet
BYHN	BY chicks fed the HN diet
BYLN	BY chicks fed the LN diet
FCR	Feed conversion ratio
ADG	Average daily gain
ADFI	Average daily feed intake
SCFA	Short-chain fatty acid
ME	Metabolizable energy
CP	Crude protein
PCR	Polymerase chain reaction
OTUs	Operational taxonomic units
PCoA	Principal coordinates analysis
