# Supplementary material for: The Interactive Effects of Nutrient Density and Breed on Growth Performance and Gut Microbiota in Broilers
Source: Animals (Basel). 2024 Dec 6;14(23):3528. doi: 10.3390/ani14233528 (PMC11639963; doi:10.3390/ani14233528)
Supplement: Supplementary file 1 [file animals-14-03528-s001.zip › animals-3297673-supplementary.pdf]

Table S1: Pairwise comparison between treatments for beta diversity of microbiome in the cecum of chickens on d42.

Pairs	Df	Sums Of Sqs	F. Model	R^2	p.value	p.adjusted
AAHN vs AALN	1	0.2037382	2.087659	0.12976771	0.014	0.028*
AAHN vs BYHN	1	0.2149846	2.362941	0.14440807	0.008	0.028*
AAHN vs BYLN	1	0.1287059	1.279659	0.08374921	0.191	0.191
AALN vs BYHN	1	0.1553055	2.360365	0.14427335	0.012	0.028*
AALN vs BYLN	1	0.1775396	2.354832	0.14398389	0.03	0.045*
BYHN vs BYLN	1	0.1397853	2.032242	0.12675971	0.041	0.0492*

AAHN: Arbor Acres broilers fed a high-nutrient diet; AALN: Arbor Acres broilers fed a low-nutrient diet; BYHN: Beijing-You chickens fed a high-nutrient diet; BYLN: Beijing-You chickens fed a low-nutrient diet. Asterisks indicate significant difference between treatment groups. Significance was set as p.adjusted value less than 0.05.
